# Synonym extraction and abbreviation expansion with ensembles of semantic spaces

**DOI:** 10.1186/2041-1480-5-6

**Published:** 2014-02-05

**Authors:** Aron Henriksson, Hans Moen, Maria Skeppstedt, Vidas Daudaravičius, Martin Duneld

**Affiliations:** 1 Department of Computer and Systems Sciences (DSV), Stockholm University, Forum 100, SE-164 40 Kista, Sweden; 2 Department of Computer and Information Science, Norwegian University of Science and Technology, NO-7491 Trondheim, Norway; 3 Faculty of Informatics, Vytautas Magnus University, Vileikos g. 8 - 409, Kaunas, LT-44404, Lithuania

## Abstract

**Background:**

Terminologies that account for variation in language use by linking synonyms and abbreviations to their corresponding concept are important enablers of high-quality information extraction from medical texts. Due to the use of specialized sub-languages in the medical domain, manual construction of semantic resources that accurately reflect language use is both costly and challenging, often resulting in low coverage. Although models of distributional semantics applied to large corpora provide a potential means of supporting development of such resources, their ability to isolate synonymy from other semantic relations is limited. Their application in the clinical domain has also only recently begun to be explored. Combining distributional models and applying them to different types of corpora may lead to enhanced performance on the tasks of automatically extracting synonyms and abbreviation-expansion pairs.

**Results:**

A combination of two distributional models – Random Indexing and Random Permutation – employed in conjunction with a single corpus outperforms using either of the models in isolation. Furthermore, combining semantic spaces induced from different types of corpora – a corpus of clinical text and a corpus of medical journal articles – further improves results, outperforming a combination of semantic spaces induced from a single source, as well as a single semantic space induced from the conjoint corpus. A combination strategy that simply sums the cosine similarity scores of candidate terms is generally the most profitable out of the ones explored. Finally, applying simple post-processing filtering rules yields substantial performance gains on the tasks of extracting abbreviation-expansion pairs, but not synonyms. The best results, measured as recall in a list of ten candidate terms, for the three tasks are: 0.39 for abbreviations to long forms, 0.33 for long forms to abbreviations, and 0.47 for synonyms.

**Conclusions:**

This study demonstrates that ensembles of semantic spaces can yield improved performance on the tasks of automatically extracting synonyms and abbreviation-expansion pairs. This notion, which merits further exploration, allows different distributional models – with different model parameters – and different types of corpora to be combined, potentially allowing enhanced performance to be obtained on a wide range of natural language processing tasks.

## Background

In order to create high-quality information extraction systems, it is important to incorporate some knowledge of semantics, such as the fact that a concept can be signified by multiple signifiers^a^. Morphological variants, abbreviations, acronyms, misspellings and synonyms – although different in form – may share semantic content to different degrees. The various lexical instantiations of a concept thus need to be mapped to some standard representation of the concept, either by converting the different expressions to a canonical form or by generating lexical variants of a concept’s 'preferred term’. These mappings are typically encoded in semantic resources, such as thesauri or ontologies^b^, which enable the recall (sensitivity) of information extraction systems to be improved. Although their value is undisputed, manual construction of such resources is often prohibitively expensive and may also result in limited coverage, particularly in the biomedical and clinical domains where language use variability is exceptionally high [1].

There is thus a need for (semi-)automatic methods that can aid and accelerate the process of lexical resource development, especially ones that are able to reflect real language use in a particular domain and adapt to different genres of text, as well as to changes over time. In the clinical domain, for instance, language use in general, and (ad-hoc) abbreviations in particular, can vary significantly across specialities. Statistical, corpus-driven and language-agnostic methods are attractive due to their inherent portability: given a corpus of sufficient size in the target domain, the methods can be applied with no or little adaptation needed. Models of distributional semantics, building on the assumption that linguistic items with similar distributions in large bodies of linguistic data have similar meanings, fulfill these requirements and have been used to extract semantically similar terms from large corpora; with increasing access to data from electronic health records, their application in the clinical domain has lately begun to be explored. In this paper, we present a method that employs distributional semantics for the extraction of synonyms and abbreviation-expansion pairs from two corpora: a clinical corpus (comprising health record narratives) and a medical corpus (comprising journal articles). We also demonstrate that performance can be enhanced by creating ensembles of (distributional) semantic spaces – both with different model parameter configurations and induced from different genres of text.

The structure of this paper is as follows. First, we present some relevant background literature on synonyms, abbreviations and their extraction/expansion. We also introduce the ideas underlying distributional semantics in general and, in particular, the models employed in this study: Random Indexing and Random Permutation. Then, we describe our method of combining semantic spaces induced from single and multiple corpora, including the details of the experimental setup and the materials used. A presentation of the experimental results is followed by an analysis and discussion of their implications. Finally, we conclude the paper with a summary and conclusions.

### Language use variability: synonyms and abbreviations

Synonymy is a semantic relation between two phonologically distinct words with very similar meaning. It is, however, extremely rare that two words have the exact same meaning – perfect synonyms – as there is often at least one parameter that distinguishes the use of one word from another [2]. For this reason, we typically speak of near-synonyms instead; that is, two words that are interchangeable in some, but not all, contexts^c^[2]. Two near-synonyms may also have different connotations, such as conveying a positive or a negative attitude. To complicate matters further, the same concept can sometimes be referred to with different words in different dialects; for a speaker who is familiar with both dialects, these can be viewed as synonyms. A similar phenomenon concerns different formality levels, where one word in a synonym pair is used only as slang and the other only in a more formal context [2]. In the medical domain, there is one vocabulary that is more frequently used by medical professionals, whereas patients often use alternative, layman terms [3]. When developing many natural language processing (NLP) applications, it is important to have ready access to terminological resources that cover this variation in the use of vocabulary by storing synonyms. Examples of such applications are query expansion [3], text simplification [4] and, as already mentioned previously, information extraction [5].

The use of abbreviations and acronyms is prevalent in both medical journal text [6] and clinical text [1]. This leads to decreased readability [7] and poses challenges for information extraction [8]. Semantic resources that also link abbreviations to their corresponding concept, or, alternatively, simple term lists that store abbreviations and their corresponding long form, are therefore as important as synonym resources for many biomedical NLP applications. Like synonyms, abbreviations are often interchangeable with their corresponding long form in some, if not all, contexts. An important difference between abbreviations and synonyms is, however, that abbreviations are semantically overloaded to a much larger extent; that is, one abbreviation often has several possible long forms, with distinct meanings. In fact, 81% of UMLS^d^ abbreviations in biomedical text were found to be ambiguous [6].

### Identifying synonymous relations between terms

The importance of synonym learning is well recognized in the NLP research community, especially in the biomedical [9] and clinical [1] domains. A wide range of techniques has been proposed for the identification of synonyms and other semantic relations, including the use of lexico-syntactic patterns, graph-based models and, indeed, distributional semantics [10] – the approach investigated in this study.

For instance, Hearst [11] proposes the use of lexico-syntactic patterns for the automatic acquisition of hyponyms^e^ from unstructured text. These patterns are hand-crafted according to observations in a corpus. Patterns can similarly be constructed for other types of lexical relations. However, a requirement is that these syntactic patterns are common enough to match a wide array of hyponym pairs. Blondel et al. [12] present a graph-based method that takes its inspiration from the calculation of hub, authority and centrality scores when ranking hyperlinked web pages. They illustrate that the central similarity score can be applied to the task of automatically extracting synonyms from a monolingual dictionary, in this case the Webster dictionary, where the assumption is that synonyms have a large overlap in the words used in their definitions; they also co-occur in the definition of many words. Another possible source for extracting synonyms is the use of linked data, such as Wikipedia. Nakayama et al. [13] also utilize a graph-based method, but instead of relying on word co-occurrence information, they exploit the links between Wikipedia articles (treated as concepts). This way they can measure both the strength (the number of paths from one article to another) and the distance (the length of each path) between concepts: concepts close to each other in the graph and with common hyperlinks are deemed to be more closely related than those farther away.

There have also been some previous efforts to obtain better performance on the synonym extraction task by not only using a single source and a single method. Inspiration for some of these approaches has been drawn from ensemble learning, a machine learning technique that combines the output of several different classifiers with the aim of improving classification performance (see [14] for an overview). Curran [15] exploits this notion for synonym extraction and demonstrates that ensemble methods outperform individual classifiers even for very large corpora. Wu and Zhou [16] use multiple resources – a monolingual dictionary, a bilingual corpus and a large monolingual corpus – in a weighted ensemble method that combines the individual extractors, thereby improving both precision and recall on the synonym acquisition task. Along somewhat similar lines, van der Plas and Tiedemann [17] use parallel corpora to calculate distributional similarity based on (automatic) word alignment, where a translational context definition is employed; synonyms are extracted with both greater precision and recall compared to a monolingual approach. This approach is, however, hardly applicable in the medical domain due to the unavailability of parallel corpora. Peirsman and Geeraerts [18] combine predictors based on collocation measures and distributional semantics with a so-called compounding approach, wherein cues are combined with strongly associated words into compounds and verified against a corpus. This ensemble approach is shown substantially to outperform the individual predictors of strong term associations in a Dutch newspaper corpus. In information retrieval, Diaz and Metzler [19] report increased performance gains when utilizing language models that derive evidence from both a target corpus and an external corpus, compared to using the target corpus alone.

In the biomedical domain, most efforts have focused on extracting synonyms of gene and protein names from the biomedical literature [20-22]. In the clinical domain, Conway and Chapman [23] propose a rule-based approach to generate potential synonyms from the BioPortal ontology – using permutations, abbreviation generation, etc. – after which candidate synonyms are verified against a large clinical corpus. Henriksson et al. [24,25] use models of distributional semantics to induce unigram word spaces and multiword term spaces from a large corpus of clinical text in an attempt to extract synonyms of varying length for SNOMED CT preferred terms. Zeng et al. [26] evaluate three query expansion methods for retrieval of clinical documents and conclude that an LDA-based topic model generates the best synonyms. Pedersen et al. [27] explore a set of measures for automatically judging semantic similarity and relatedness among medical term pairs that have been pre-assessed by human experts. The measures range from ones based on thesauri or ontologies (WordNet, SNOMED-CT, UMLS, Mayo Clinic Thesaurus) to those based on distributional semantics. They find that the measure based on distributional semantics performs at least as good as any of the ontology-dependent measures. In a similar task, Koopman et al. [28] evaluate eight different data-driven measures of semantic similarity. Using two separate training corpora, one containing clinical notes and the other medical literature articles, they conclude that the choice of training corpus has a significant impact on the performance of these measures.

### Creating abbreviation dictionaries automatically

There are a number of studies on the automatic creation of biomedical abbreviation dictionaries that exploit the fact that abbreviations are sometimes defined in the text on their first mention. These studies extract candidates for abbreviation-expansion pairs by assuming that either the long form or the abbreviation is written in parentheses [29]; other methods that use rule-based pattern matching have also been proposed [30]. The process of determining which of the extracted candidates that are likely to be correct abbreviation-expansion pairs is then performed either by rule-based [30] or machine learning [31,32] methods. Most of these studies have been conducted for English; however, there is also a study on Swedish medical text [33], for instance.

Yu et al. [34] have, however, found that around 75% of all abbreviations found in biomedical articles are never defined in the text. The application of these methods to clinical text is most likely inappropriate, as clinical text is often written in a telegraphic style, mainly for documentation purposes [1]; that effort would be spent on defining used abbreviations in this type of text seems unlikely. There has, however, been some work on identifying such undefined abbreviations [35], as well as on finding the intended abbreviation expansion among several possible expansions available in an abbreviation dictionary [36].

In summary, automatic creation of biomedical abbreviation dictionaries from texts where abbreviations are defined is well studied. This is also the case for abbreviation disambiguation given several possible long forms in an abbreviation dictionary. The abbreviation part of this study, however, focuses on a task that has not as yet been adequately explored: to find abbreviation-expansion pairs without requiring the abbreviations to be defined in the text.

### Distributional semantics: inducing semantic spaces from corpora

Distributional semantics (see [37] for an overview of methods and their application in the biomedical domain) were initially motivated by the inability of the vector space model [38] – as it was originally conceived – to account for the variability of language use and word choice stemming from natural language phenomena such as synonymy. To overcome the negative impact this had on recall in information retrieval systems, models of distributional semantics were proposed [39-41]. The theoretical foundation underpinning such semantic models is the *distributional hypothesis*[42], which states that words with similar distributions in language – in the sense that they co-occur with overlapping sets of words – tend to have similar meanings. Distributional methods have become popular with the increasing availability of large corpora and are attractive due to their computational approach to semantics, allowing an estimate of the semantic relatedness between two terms to be quantified.

An obvious application of distributional semantics is the extraction of semantically related terms. As near-synonyms are interchangeable in at least some contexts, their distributional profiles are likely to be similar, which in turn means that synonymy is a semantic relation that should, to a certain degree, be captured by these methods. This seems intuitive, as, next to identity, the highest degree of semantic relatedness between terms is realized by synonymy. It is, however, well recognized that other semantic relations between terms that share similar contexts will likewise be captured by these models [43]; synonymy cannot readily be isolated from such relations.

Spatial models^f^ of distributional semantics generally differ in how vectors representing term meaning are constructed. These vectors, often referred to as *context vectors*, are typically derived from a term-context matrix that contains the (weighted, normalized) frequency with which terms occur in different contexts. Working directly with such high-dimensional (and inherently sparse) data — where the dimensionality is equal to the number of contexts (e.g. the number of documents or the size of the vocabulary, depending on which context definition is employed) — would entail unnecessary computational complexity, in particular since most terms only occur in a limited number of contexts, which means that most cells in the matrix will be zero. The solution is to project the high-dimensional data into a lower-dimensional space, while approximately preserving the relative distances between data points. The benefit of dimensionality reduction is two-fold: on the one hand, it reduces complexity and data sparseness; on the other hand, it has also been shown to improve the coverage and accuracy of term-term associations, as, in this reduced (semantic) space, terms that do not necessarily co-occur directly in the *same* contexts – this is indeed the typical case for synonyms and abbreviation-expansion pairs – will nevertheless be clustered about the same subspace, as long as they appear in *similar* contexts, i.e. have neighbors in common (co-occur with the same terms). In this way, the reduced space can be said to capture higher order co-occurrence relations.

In latent semantic analysis (LSA) [39], dimensionality reduction is performed with a computationally expensive matrix factorization technique known as singular value decomposition. Despite its popularity, LSA has consequently received some criticism for its poor scalability properties. More recently, alternative methods for constructing semantic spaces based on term co-occurrence information have been proposed.

#### Random indexing

Random indexing (RI) [44] is an incremental, scalable and computationally efficient alternative to LSA in which explicit dimensionality reduction is avoided^g^: a lower dimensionality *d* is instead chosen *a priori* as a model parameter and the *d*-dimensional context vectors are then constructed incrementally. This approach allows new data to be added at any given time without having to rebuild the semantic space. RI can be viewed as a two-step operation: 

1. Each *context* (e.g. each document or unique term) is first given a static, unique representation in the vector space that is approximately uncorrelated to all other contexts. This is achieved by assigning a sparse, ternary^h^ and randomly generated *d*-dimensional *index vector*: a small number (usually around 1–2%) of +1’s and -1’s are randomly distributed, with the rest of the elements set to zero. By generating sparse vectors of a sufficiently high dimensionality in this way, the index vectors will be *nearly* orthogonal^i^.

2. Each *unique term* is assigned an initially empty *context vector* of the same dimensionality *d*. The context vectors are then incrementally populated with context information by adding the (weighted) index vectors of the contexts in which the target term appears. With a sliding window context definition, this means that the index vectors of the surrounding terms are added to the target term’s context vector. The meaning of a term, represented by its context vector, is effectively the (weighted) sum of all the contexts in which it occurs.

#### Random permutation

Models of distributional semantics, including RI, generally treat each context as a *bag of words*^j^. Such models are often criticized for failing to account for term order. Recently, methods have been developed for building distributional semantic models that store and emphasize word order information [45-47]. Random permutation (RP) [46] is a modification of RI that encodes term order information by simply *permuting* (i.e., shifting) the elements in the index vectors according to their direction and distance^k^ from the target term before they are added to the context vector. For instance, before adding the index vector of a term two positions to the left of the target term, the elements are shifted two positions to the left; similarly, before adding the index vector of a term one position to the right of the target term, the elements are shifted one position to the right. In effect, each term has multiple unique representations: one index vector for each possible position relative to the target term in the context window. Incorporating term order information not only enables order-based retrieval; it also constrains the types of semantic relations that are captured.

#### Model parameters

There are a number of model parameters that need to be configured according to the task that the induced semantic spaces will be used for. For instance, the types of semantic relations captured depends on the context definition [43,48]. By employing a document-level context definition, relying on direct co-occurrences, one models *syntagmatic* relations. That is, two terms that frequently co-occur in the same documents are likely to be about the same general topic. By employing a sliding window context definition, one models *paradigmatic* relations. That is, two terms that frequently co-occur with similar sets of words – i.e., share neighbors – but do not necessarily co-occur themselves, are semantically similar. Synonymy is a prime example of a paradigmatic relation. The size of the context window also affects the types of relations that are modeled and needs to be tuned for the task at hand. This is also true for semantic spaces produced by RP; however, the precise impact of window size on RP spaces and the internal relations of their context vectors is yet to be studied in depth.

## Method

**Figure 1:**
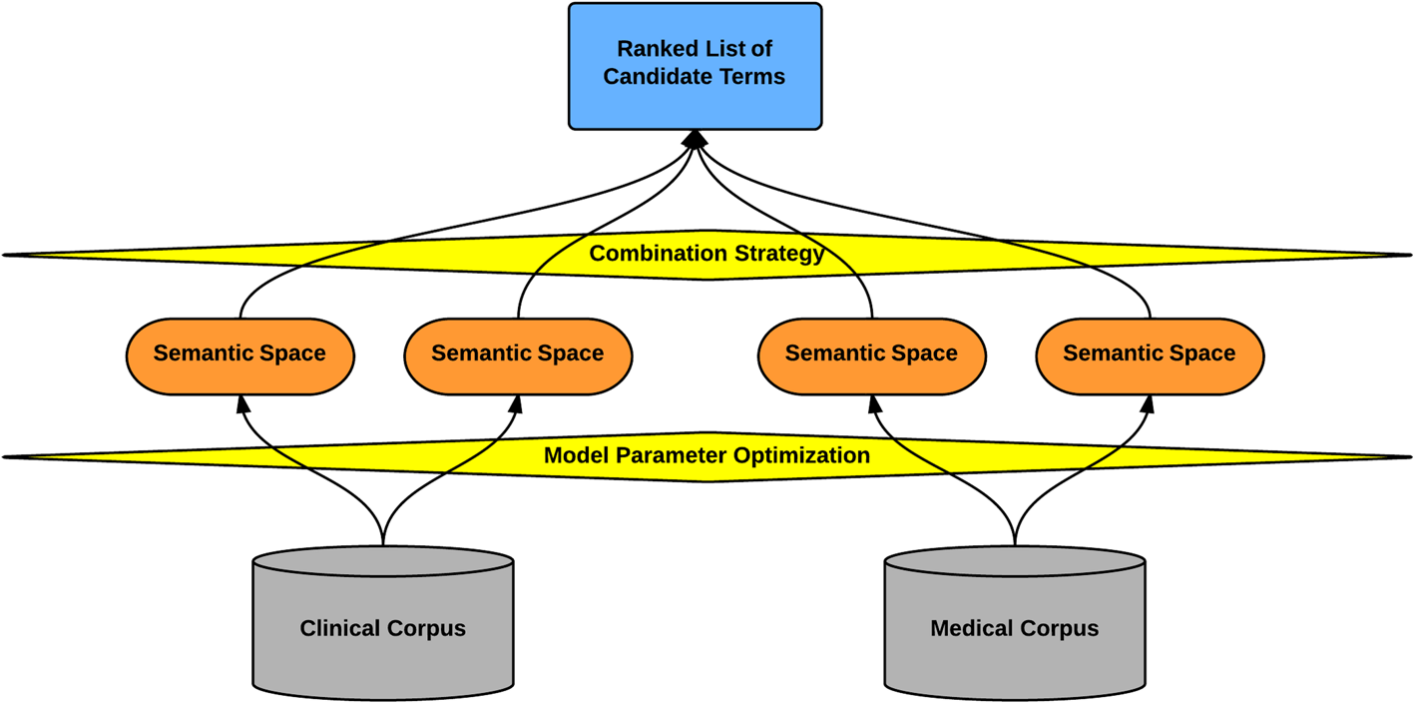
**Ensembles of semantic spaces for synonym extraction and abbreviation expansion.** Semantic spaces built with different model parameters are induced from different corpora. The output of the semantic spaces are combined in order to obtain better results compared to using a single semantic space in isolation.

The main idea behind this study is to enhance the performance on the task of extracting synonyms and abbreviation-expansion pairs by combining multiple and different semantic spaces – different in terms of (1) type of model and model parameters used, and (2) type of corpus from which the semantic space is induced. In addition to combining semantic spaces induced from a single corpus, we also combine semantic spaces induced from two different types of corpora: in this case, a clinical corpus (comprising health record notes) and a medical corpus (comprising journal articles). The notion of combining multiple semantic spaces to improve performance on some task is generalizable and can loosely be described as creating *ensembles of semantic spaces*. By combining semantic spaces, it becomes possible to benefit from model types that capture slightly different aspects of semantics, to exploit various model parameter configurations (which influence the types of semantic relations that are modeled), as well as to observe language use in potentially very different contexts (by employing more than one corpus type). We set out exploring this approach by querying each semantic space separately and then combining their output using a number of combination strategies (Figure 1).

The experimental setup can be divided into the following steps: (1) corpora preprocessing, (2) construction of semantic spaces from the two corpora (and from the conjoint corpus), (3) identification of the most profitable single-corpus (and conjoint corpus) combinations, (4) identification of the most profitable (disjoint) multiple-corpora combinations, (5) evaluations of the single-corpus (including the conjoint corpus) and multiple-corpora combinations, (6) post-processing of candidate terms, and (7) frequency threshold experiments. Once the corpora have been preprocessed, ten semantic spaces from each corpus, as well as the conjoint corpus, are induced with different context window sizes (RP spaces are induced with and without stop words). Ten pairs of semantic spaces are then combined using three different combination strategies. These are evaluated on the three tasks – (1) abbreviations → expansions, (2) expansions → abbreviations and (3) synonyms – using the development subsets of the reference standards (a list of medical abbreviation-expansion pairs for 1 and 2 and MeSH synonyms for 3). Performance is mainly measured as recall top 10, i.e. the proportion of expected candidate terms that are among a list of ten suggestions. The pair of semantic spaces involved in the most profitable combination for each corpus is then used to identify the most profitable multiple-corpora combinations, where eight different combination strategies are evaluated. The best single-corpus combinations are evaluated on the evaluation subsets of the reference standards, where using RI and RP in isolation constitute the two baselines. The best multiple-corpora combination is likewise evaluated on the evaluation subsets of the reference standards; here, the results are compared both to (1) semantic spaces induced from a single corpus and the conjoint corpus, and (2) ensembles of semantic spaces induced from a single corpus (and the conjoint corpus). Post-processing rules are then constructed using the development subsets of the reference standards and the outputs of the various semantic space combinations. These are evaluated on the evaluation subsets of the reference standards using the most profitable single-corpus and multiple-corpora ensembles. All evaluations on the evaluation subsets of the reference standards also include an evaluation of weighted precision, see Eq. 1:

(1) $$\begin{array}{ll} \text{Weighted Precision:} & P_{w}=\frac{\overset{j-1}{\underset{i=0}{\sum }}(j-i)\cdot f(i)}{\overset{j-1}{\underset{i=0}{\sum }}j-i}\\ & \text{where}\\ & f(i)=\left\{\begin{array}{cc} 1 & \text{if}\;i\;\in \;\{\text{tp}\}\\ 0 & \text{otherwise} \end{array}\right) \end{array}$$

and *j* is the pre-specified number of labels – here, ten, except in the case of a dynamic cut-off – and {*tp*} is the set of true positives. In words, this assigns a score to true positives according to their (reverse) ranking in the list, sums their scores and divides the total score by the maximum possible score (where all *j* labels are true positives).

Finally, we explore the impact of frequency thresholds (i.e., how many times each pair of terms in the reference standards needs to occur to be included) on performance.

### Inducing semantic spaces from clinical and medical corpora

Each individual semantic space is constructed with one model type, using a predefined context window size and induced from a single corpus type. The semantic spaces are constructed with random indexing (RI) and random permutation (RP) using JavaSDM [49]. For all semantic spaces, a dimensionality of 1,000 is used (with 8 non-zero, randomly distributed elements in the index vectors: four 1s and four -1s). When the RI model is employed, the index vectors are weighted according to their distance from the target term, see Eq. 2, where *dist*_*it*_ is the distance to the target term. When the RP model is employed, the elements of the index vectors are instead shifted according to their direction and distance from the target term; no weighting is performed.

(2) $${\text{weight}}_{i}=2^{1-{\text{dist}}_{\text{it}}}$$

For all models, window sizes of two (1 + 1), four (2 + 2) and eight (4 + 4) surrounding terms are used. In addition, RI spaces with a window size of twenty (10 + 10) are induced in order to investigate whether a significantly wider context definition may be profitable. Incorporating order information (RP) with such a large context window makes little sense; such an approach would also suffer from data sparseness. Different context definitions are experimented with in order to find one that is best suited to each task. The RI spaces are induced only from corpora that have been stop-word filtered, as co-occurrence information involving high-frequent and widely distributed words contribute very little to the meaning of terms. The RP spaces are, however, also induced from corpora in which stop words have been retained. The motivation behind this is that all words, including function words – these make up the majority of the items in the stop-word lists – are important to the syntactic structure of language and may thus be of value when modeling order information [45]. A stop-word list is created for each corpus by manually inspecting the most frequent word types and removing from the list those words that may be of interest, e.g. domain-specific terms. Each list consists of approximately 150 terms.

The semantic spaces are induced from two types of corpora – essentially belonging to different genres, but both within the wider domain of medicine: (1) a clinical corpus, comprising notes from health records, and (2) a medical corpus, comprising medical journal articles.

The *clinical corpus* contains a subset of the Stockholm EPR Corpus [50], which encompasses health records from the Karolinska University Hospital in Stockholm, Sweden over a five-year period^l^. The clinical corpus used in this study is created by extracting the free-text, narrative parts of the health records from a wide range of clinical practices. The clinical notes are written in Swedish by physicians, nurses and other health care professionals over a six-month period in 2008. In summary, the corpus comprises documents that each contain clinical notes documenting a single patient visit at a particular clinical unit.

The *medical corpus* contains the freely available subset of Läkartidningen (1996–2005), which is the Journal of the Swedish Medical Association [51]. It is a weekly journal written in Swedish and contains articles discussing new scientific findings in medicine, pharmaceutical studies, health economic evaluations, etc. Although these issues have been made available for research, the original order of the sentences has not been retained due to copyright reasons. The sentences thus appear in a randomized order, which means that the original texts cannot be recreated.

Both corpora are lemmatized using the *Granska Tagger*[52] and thereafter further preprocessed by removing punctuation marks and digits. Two versions of each corpus are created: one version in which the stop words are retained and one version in which they are removed^m^. As the sentences in Läkartidningen are given in a random order, a document break is indicated between each sentence for this corpus. It is thereby ensured that context information from surrounding sentences will not be incorporated in the induced semantic space. Statistics for the two corpora are shown in Table 1.

**Table 1:** Corpora statistics

**Corpus**	**With stop words**	**Without stop words**	**Segments**
Clinical	∼42.5M tokens	∼22.5M tokens	268,727 documents
	(∼0.4M types)	(∼0.4M types)	
Medical	∼20.3M tokens	∼12.1M tokens	1,153,824 sentences
	(∼0.3M types)	(∼0.3M types)	

The number of tokens and unique terms (word types) in the medical and clinical corpus, with and without stop words.

In summary, a total of thirty semantic spaces are induced – ten from each corpus type, and ten from the conjoint corpus. Four RI spaces are induced from each corpus type (12 in total), the difference being the context definition employed (1 + 1, 2 + 2, 4 + 4, 10 + 10). Six RP spaces are induced from each corpus type (18 in total), the difference being the context definition employed (1 + 1, 2 + 2, 4 + 4) and whether stop words have been removed or retained (sw).

### Combinations of semantic spaces from a single corpus

Since RI and RP model semantic relations between terms in slightly different ways, it may prove profitable to combine them in order to increase the likelihood of capturing synonymy and identifying abbreviation-expansion pairs. In one study it was estimated that the overlap in the output produced by RI and RP spaces is, on average, only around 33% [46]: by combining them, we hope to capture different semantic properties of terms and, ultimately, boost results. The combinations from a single corpus type involve only two semantic spaces: one constructed with RI and one constructed with RP. In this study, the combinations involve semantic spaces with identical window sizes, with the following exception: RI spaces with a wide context definition (10 + 10) are combined with RP spaces with a narrow context definition (1 + 1, 2 + 2). The RI spaces are combined with RP spaces both with and without stop words.

Three different strategies of combing an RI-based semantic space with an RP space are designed and evaluated. Thirty combinations are evaluated for each corpus, i.e. sixty in total (Table 2). The three combination strategies are: 

•*RI*⊂*RP*_30_

•Finds the top ten terms in the RI space that are among the top thirty terms in the RP space.

•*RP*⊂*RI*_30_

•Finds the top ten terms in the RP space that are among the top thirty terms in the RI space.

•*RI*+*RP*

•Sums the cosine similarity scores from the two spaces for each candidate term.

**Table 2:** Overview of experiments conducted with a single semantic space

For each of the **2** corpora, **10** semantic spaces were induced.							
**RI spaces**	RI_20	RI_2		RI_4		RI_8	
**RP spaces**		RP_2	RP_2_sw	RP_4	RP_4_sw	RP_8	RP_8_sw
The induced semantic spaces were combined in **10** different combinations.							
**Combinations**							
Identical window size		RI_2, RP_2		RI_4, RP_4		RI_8, RP_8	
Identical window size, stop words		RI_2, RP_2_sw		RI_4, RP_4_sw		RI_8, RP_8_sw	
Large window size		RI_20, RP_2		RI_20, RP_4			
Large window size, stop words		RI_20, RP_2_sw		RI_20, RP_4_sw			
For each combination, **3** combination strategies were evaluated.							
**Combination strategies**	*RI* ⊂ *RP*_30_		*RP* ⊂ *RI*_30_		*RI* + *RP*		

For each of the two corpora and the conjoint corpus, 30 different combinations were evaluated. The configurations are described according to the following pattern: *model_windowSize*. For RP, *sw* means that stop words are retained in the semantic space. For instance, *model_20* means a window size of 10+10 was used.

For the first two strategies (*RI*⊂*RP*_30_ and *RP*⊂*RI*_30_) a two-stage approach is applied. First one type of model is used (RI or RP) to produce an initial ranking of words according to a given query. The other model type, trained on the same corpus, is then used to re-rank the top 30 words produced by the first model according to its internal ranking. The intuition behind this approach is to see if synonyms and abbreviation-expansion pairs can be detected by trying to ensure that the set of contextually related words also have similar grammatical properties, and vice versa. In the third strategy (*RI*+*RP*), we apply a straightforward summing of the generated similarity scores.

### Combinations of semantic spaces from multiple corpora

In addition to combining semantic spaces induced from one and the same corpus, a combination of semantic spaces induced from multiple corpora could potentially yield even better performance on the task of extracting synonyms and abbreviation-expansion pairs, especially if the terms of interest occur with some minimum frequency in both corpora. Such ensembles of semantic spaces – in this study consisting of four semantic spaces – allow not only different model types and model parameter configurations to be employed, but also allow us to capture language use in different genres or domains, in which terms may be used in slightly different contexts. The pair of semantic spaces from each corpus that is best able to perform each of the aforementioned tasks – consisting of two semantic spaces – is subsequently combined using various combination strategies.

The combination strategies can usefully be divided into two sets of approaches: in the first, the four semantic spaces are treated equally – irrespective of source – and combined in a single step; in the other, a two-step approach is assumed, wherein each pair of semantic spaces – induced from the same source – is combined separately before the combination of combinations is performed. In both sets of approaches, the outputs of the semantic spaces are combined in one of two ways: *SUM*, where the cosine similarity scores are merely summed, and *AVG*, where the average cosine similarity score is calculated based on the number of semantic spaces in which the term under consideration exists. The latter is an attempt to mitigate the effect of differences in vocabulary between the two corpora. In the two-step approaches, the *SUM*/*AVG* option is configurable for each step. In the single-step approaches, the combinations can be performed either with or without *normalization*, which in this case means replacing the exact cosine similarity scores of the candidate terms in the output of each queried semantic space with their ranking in the list of candidate terms. This means that the candidate terms are now sorted in ascending order, with zero being the highest score. When combining two or more lists of candidate terms, the combined list is also sorted in ascending order. The rationale behind this option is that the cosine similarity scores are relative and thus only valid within a given semantic space: combining similarity scores from semantic spaces constructed with different model types and parameter configurations, and induced from different corpora, might have adverse effects. In the two-step approach, normalization is always performed after combining each pair of semantic spaces. In total, eight combination strategies are evaluated:

#### Single-step approaches

•SUM: *RI*_*clinical*_+*RP*_*clinical*_+*RI*_*medical*_+*RP*_*medical*_

•Each candidate term’s cosine similarity score in each semantic space is summed. The top ten terms from this list are returned.

•SUM, normalized: *norm*(*RI*_*clinical*_)+*norm*(*RP*_*clinical*_)+*norm*(*RI*_*medical*_)+*norm*(*RP*_*medical*_)

•The output of each semantic space is first normalized by using the ranking instead of cosine similarity; each candidate term’s (reverse) ranking in each semantic space is then summed. The top ten terms from this list are returned.

•AVG: $\frac{{\text{RI}}_{\text{clinical}}+{\text{RP}}_{\text{clinical}}+{\text{RI}}_{\text{medical}}+{\text{RP}}_{\text{medical}}}{{\text{count}}_{\text{term}}}$

•Each candidate term’s cosine similarity score in each semantic space is summed; this value is then averaged over the number of semantic spaces in which the term exists. The top ten terms from this list are returned.

•AVG, normalized: $\frac{\text{norm}({\text{RI}}_{\text{clinical}})\;+\;\text{norm}({\text{RP}}_{\text{clinical}})\;+\;\text{norm}({\text{RI}}_{\text{medical}})\;+\;\text{norm}({\text{RP}}_{\text{medical}})}{{\text{count}}_{\text{term}}}$

•The output of each semantic space is first normalized by using the ranking instead of cosine similarity; each candidate term’s normalized score in each semantic space is then summed; this value is finally averaged over the number of semantic spaces in which the term exists. The top ten terms from this list are returned.

#### Two-step approaches

•SUM →SUM: *norm*(*RI*_*clinical*_+*RP*_*clinical*_)+*norm*(*RI*_*medical*_+*RP*_*medical*_)

•Each candidate term’s cosine similarity score in each pair of semantic spaces is first summed; these are then normalized by using the ranking instead of the cosine similarity; finally, each candidate term’s normalized score is summed. The top ten terms from this list are returned.

•AVG →AVG: $\frac{\text{norm}\;\left(\;\frac{{\text{RI}}_{\text{clinical}}\;+\;{\text{RP}}_{\text{clinical}}}{{\text{count}}_{\text{term}-\text{source}-a}}\;\right)\;+\;\text{norm}\left(\;\frac{{\text{RI}}_{\text{medical}}\;+\;{\text{RP}}_{\text{medical}}}{{\text{count}}_{\text{term}-\text{source}-b}}\;\right)}{{\text{count}}_{\text{term}-\text{source}-a}\;+\;{\text{count}}_{\text{term}-\text{source}-b}}$

•Each candidate term’s cosine similarity score for each pair of semantic spaces is first summed; for each pair of semantic spaces, this value is then averaged over the number of semantic spaces in that pair in which the term exists; these are subsequently normalized by using the ranking instead of the cosine similarity; each candidate term’s normalized score in each combined list is then summed and averaged over the number of semantic spaces in which the term exists (in both pairs of semantic spaces). The top ten terms from this list are returned.

•SUM →AVG: $\frac{\text{norm}({\text{RI}}_{\text{clinical}}\;+\;{\text{RP}}_{\text{clinical}})\;+\;\text{norm}({\text{RI}}_{\text{medical}}\;+\;{\text{RP}}_{\text{medical}})}{{\text{count}}_{\text{term}}}$

•Each candidate term’s cosine similarity score for each pair of semantic spaces is first summed; these are then normalized by using the ranking instead of the cosine similarity; each candidate term’s normalized score in each combined list is then summed and averaged over the number of semantic spaces in which the term exists. The top ten terms from this list are returned.

•AVG →SUM: $\text{norm}\left(\frac{{\text{RI}}_{\text{clinical}}+{\text{RP}}_{\text{clinical}}}{{\text{count}}_{\text{term}}}\right)+\text{norm}\left(\frac{{\text{RI}}_{\text{medical}}+{\text{RP}}_{\text{medical}}}{{\text{count}}_{\text{term}}}\right)$

•Each candidate term’s cosine similarity score for each pair of semantic spaces is first summed and averaged over the number of semantic spaces in that pair in which the term exists; these are then normalized by using the ranking instead of the cosine similarity; each candidate term’s normalized score in each combined list is finally summed. The top ten terms from this list are returned.

### Post-processing of candidate terms

In addition to creating ensembles of semantic spaces, simple filtering rules are designed and evaluated for their ability to enhance performance further on the task of extracting synonyms and abbreviation-expansion pairs. For obvious reasons, this is easier for abbreviation-expansion pairs than for synonyms.

With regards to abbreviation-expansion pairs, the focus is on increasing precision by discarding poor suggestions in favor of potentially better ones. This is attempted by exploiting properties of the abbreviations and their corresponding expansions. The development subset of the reference standard (see Evaluation framework) is used to construct rules that determine the validity of candidate terms. For an abbreviation-expansion pair to be considered valid, each letter in the abbreviation has to be present in the expansion and the letters also have to appear in the same order. Additionally, the length of abbreviations and expansions is restricted, requiring an expansion to contain more than four letters, whereas an abbreviation is allowed to contain a maximum of four letters. These rules are shown in Eq. 3 and Eq. 4.

**Figure 2:**
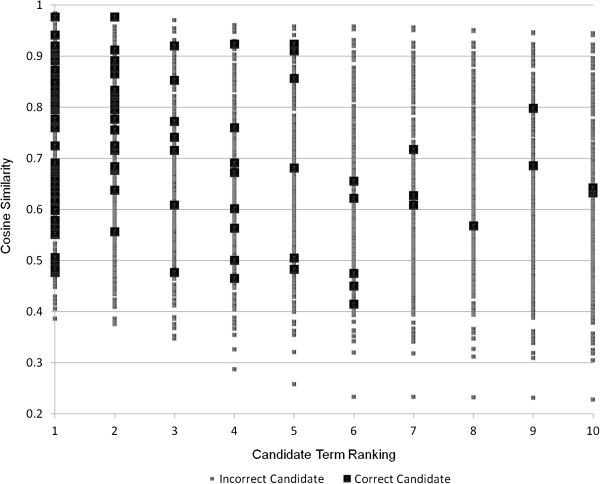
**Distribution of candidate terms for the clinical corpus.** The distribution (cosine similarity and rank) of candidates for synonyms for the best combination of semantic spaces induced from the clinical corpus. The results show the distribution for query terms in the development reference standard.

**Figure 3:**
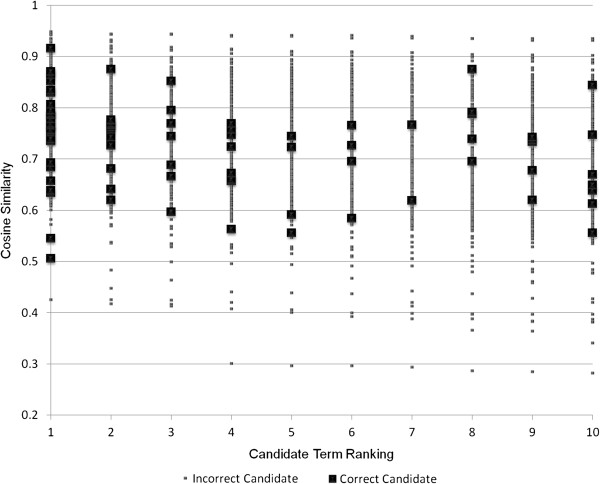
**Distribution of candidate terms for the medical corpus.** The distribution (cosine similarity and rank) of candidates for synonyms for the best combination of semantic spaces induced from the medical corpus. The results show the distribution for query terms in the development reference standard.

**Figure 4:**
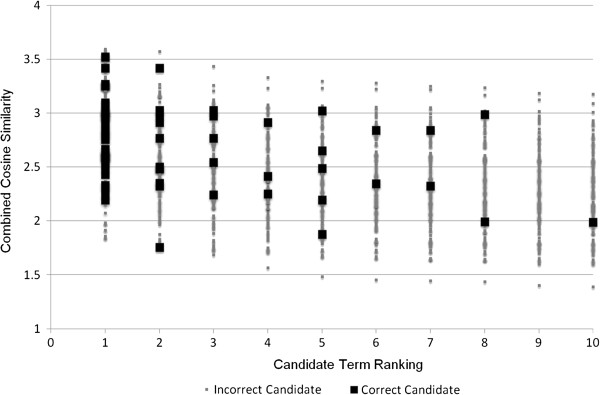
**Distribution of candidate terms for clinical + medical corpora.** The distribution (combined cosine similarity and rank) of candidates for synonyms for the ensemble of semantic spaces induced from medical and clinical corpora. The results show the distribution for query terms in the development reference standard.

For synonym extraction, cut-off values for rank and cosine similarity are instead employed. These cut-off values are tuned to maximize precision for the best semantic space combinations in the development subset of the reference standard, without negatively affecting recall (see Figures 2, 3 and 4). Used cut-off values are shown in Eq. 5 for the clinical corpus, in Eq. 6 for the medical corpus, and in Eq. 7 for the combination of the two corpora. In Eq. 7, *Cos* denotes the combination of the cosine values, which means that it has a maximum value of four rather than one.

(3) $$\;\text{Exp}\to \text{Abbr}=\left\{\begin{array}{ll} \text{True}, & \text{if}\;(\text{Len}<5)\wedge ({\text{Sub}}_{\text{out}}=\text{True})\\ \text{False}, & \text{Otherwise} \end{array}\right)$$

(4) $$\;\text{Abbr}\to \text{Exp}=\left\{\begin{array}{ll} \text{True}, & \text{if}\;(\text{Len}>4)\wedge ({\text{Sub}}_{\text{in}}=\text{True})\\ \text{False}, & \text{Otherwise} \end{array}\right)$$

(5) $$\;{\text{Syn}}_{\text{clinical}}\;=\;\;\left\{\begin{array}{l} \;\;\;\;\text{True},\;\text{if}\;(\text{Cos}\;\geq \;0.60)\;\vee \;(\text{Cos}\;\geq \;0.40\;\wedge \;\text{Rank}\;<\;9)\\ \;\;\;\;\text{False},\;\text{Otherwise} \end{array}\right)$$

(6) $${\text{Syn}}_{\text{medical}}=\left\{\begin{array}{ll} \text{True}, & \text{if}\;(\text{Cos}\geq 0.50)\\ \text{False}, & \text{Otherwise} \end{array}\right)$$

(7) $${\text{Syn}}_{\text{clinical}+\text{medical}}=\left\{\begin{array}{ll} \text{True}, & \text{if}\;(\text{Cos}\geq 1.9)\vee (\text{Cos}\geq 1.8\wedge \text{Rank}<6)\vee (\text{Cos}\geq 1.75\wedge \text{Rank}<3)\\ \text{False}, & \text{Otherwise} \end{array}\right)$$

Cos: Cosine similarity between candidate term and query term.

Rank: The ranking of the candidate term, ordered by cosine similarity.

*Sub*_*out*_*: Whether each letter in the candidate term is present in the query term, in the same order and with identical initial letters.*

*Sub*_*in*_*: Whether each letter in the query term is present in the candidate term, in the same order and with identical initial letters.*

Len: The length of the candidate term.

The post-processing filtering rules are employed in two different ways. In the first approach, the semantic spaces are forced to suggest a predefined number of candidate terms (ten), irrespective of how good they are deemed to be by the semantic space. Candidate terms are retrieved by the semantic space until ten have been classified as correct according to the post-processing rules, or until one hundred candidate terms have been classified. If less than ten are classified as incorrect, the highest ranked discarded terms are used to populate the remaining slots in the final list of candidate terms. In the second approach, the semantic spaces are allowed to suggest a dynamic number of candidate terms, with a minimum of one and a maximum of ten. If none of the highest ranked terms are classified as correct, the highest ranked term is suggested.

### Evaluation framework

Evaluation of the numerous experiments is carried out with the use of reference standards: one contains known abbreviation-expansion pairs and the other contains known synonyms. The semantic spaces and their various combinations are evaluated for their ability to extract known abbreviations/expansions (*abbr* →*exp* and *exp* →*abbr*) and synonyms (*syn*) – according to the employed reference standard – for a given query term in a list of ten candidate terms (recall top 10). Recall is prioritized in this study and any decisions, such as deciding which model parameters or which combination strategies are the most profitable, are solely based on this measure. When precision is reported, it is calculated as weighted precision, where the weights are assigned according to the ranking of a correctly identified term.

The reference standard for abbreviations is taken from Cederblom [53], which is a book that contains lists of medical abbreviations and their corresponding expansions. These abbreviations have been manually collected from Swedish health records, newspapers, scientific articles, etc. For the synonym extraction task, the reference standard is derived from the freely available part of the Swedish version of MeSH [54] – a part of UMLS – as well as a Swedish extension that is not included in UMLS [55]. As the semantic spaces are constructed only to model unigrams, all multiword expressions are removed from the reference standards. Moreover, hypernym/hyponym and other non-synonym pairs found in the UMLS version of MeSH are manually removed from the reference standard for the synonym extraction task. Models of distributional semantics sometimes struggle to model the meaning of rare terms accurately, as the statistical basis for their representation is insufficiently solid. As a result, we only include term pairs that occur at least fifty times in each respective corpus. This, together with the fact that term frequencies differ from corpus to corpus, means that one separate reference standard is used for the evaluation of the clinical corpus and another is used for the evaluation of the medical corpus. For evaluating combinations of semantic spaces induced from different corpora, a third – common – reference standard is therefore created, in which only term pairs that occur at least fifty times in both corpora are included. Included terms are not restricted to form pairs; in the reference standard for the synonym extraction task, some form larger groups of terms with synonymous relations. There are also abbreviations with several possible expansions, as well as expansions with several possible abbreviations. The term pairs (or n-tuples) in each reference standard are randomly split into a *development set* and an *evaluation set* of roughly equal size. The development sets are used for identifying the most profitable ensembles of semantic spaces (with optimized parameter settings, such as window size and whether to include stop words in the RP spaces) for each of the three tasks, as well as for creating the post-processing filtering rules. The evaluation sets are used for the final evaluation to assess the expected performance of the ensembles in a deployment setting. Baselines for the single-corpus ensembles are created by employing RI and RP in isolation; baselines for the multiple-corpora ensembles are created by using the most profitable clinical and medical ensembles from the single-corpus experiments, as well a single space induced from the conjoint corpus and an ensemble of semantic spaces induced from the conjoint corpus. Statistics for the reference standards are shown in Table 3. The differences in recall between the different semantic spaces/ensembles, when evaluated on the evaluation subset of the reference standards, are tested for statistical significance. The exact binomial sign test is used ([56], pp. 532–535), assuming independence between all query terms.

**Table 3:** Reference standards statistics

**Reference standard**	**Clinical corpus**			**Medical corpus**			**Clinical + Medical**		
	**Size**	**2 Cor**	**3 Cor**	**Size**	**2 Cor**	**3 Cor**	**Size**	**2 Cor**	**3 Cor**
Abbr →Exp (Devel)	117	9.4%	0.0%	55	13%	1.8%	42	14%	0%
Abbr →Exp (Eval)	98	3.1%	0.0%	55	11%	0%	35	2.9%	0%
Exp →Abbr (Devel)	110	8.2%	1.8%	63	4.7%	0%	45	6.7%	0%
Exp →Abbr (Eval)	98	7.1%	0.0%	61	0%	0%	36	0%	0%
Syn (Devel)	334	9.0%	1.2%	266	11%	3.0%	122	4.9%	0%
Syn (Eval)	340	14%	2.4%	263	13%	3.8%	135	11%	0%

*Size* shows the number of queries, *2 cor* shows the proportion of queries with two correct answers and *3 cor* the proportion of queries with three (or more) correct answers. The remaining queries have one correct answer.

In addition to the automatic evaluation using the reference standards, a small manual evaluation is also carried out on the synonym task. A random sample of 30 query terms (out of 135 terms in the *Clinical + Medical* reference standard) and their respective ten candidate terms as suggested by the best combination of semantic spaces is investigated and a manual classification of the semantic relation between each of the candidate terms and the target term is carried out. The candidate terms are manually classified as either a synonym, an antonym^n^, a hypernym^o^, a hyponym or an alternative spelling (for instance *rinitis/rhinitis*) of the target term.

## Results

The experimental setup was designed in such a manner that the semantic spaces that performed best in combination for a single corpus would also be used in the subsequent combinations from multiple corpora. Identifying the most profitable combination strategy for each of the three tasks was achieved using the development subsets of the reference standards. These combinations were then evaluated on separate evaluation sets containing unseen data. All further experiments, including the post-processing of candidate terms, were carried out with these combinations on the evaluation sets. This is therefore also the order in which the results will be presented.

### Combination strategies: a single corpus

The first step involved identifying the most appropriate window sizes for each task, in conjunction with evaluating the combination strategies. The reason for this is that the optimal window sizes for RI and RP in isolation are not necessarily identical to the optimal window sizes when RI and RP are combined. In fact, when RI is used in isolation, a window size of 2 + 2 performs best on the two abbreviation-expansion tasks, and a window size of 10 + 10 performs best on the synonym task. For RP, a semantic space with a window size of 2 + 2 yields the best results on two of the tasks – *abbr* →*exp* and *syn* – while a window size of 4 + 4 is more successful on the *exp* →*abbr* task. These are the model configurations used in the RI and RP baselines, to which the single-corpus combination strategies are compared in the final evaluation.

Using the semantic spaces induced from the clinical corpus, the *RI*+*RP* combination strategy, wherein the cosine similarity scores are merely summed, is the most successful on all three tasks: 0.42 recall on the *abbr* →*exp* task, 0.32 recall on the *exp* →*abbr* task, and 0.40 recall on the *syn* task (Table 4). For the abbreviation expansion task, a window size of 2 + 2 appears to work well for both models, with the RP space retaining stop words. On the task of identifying the abbreviated form of an expansion, semantic spaces with window sizes of 2 + 2 and 4 + 4 perform equally well; the RP spaces should include stop words. Finally, on the synonym extraction task, an RI space with a large context window (10 + 10) in conjunction with an RP space with stop words and a window size of 2 + 2 is the most profitable.

**Table 4:** Results on clinical development set

**Strategy**	**Abbr →Exp**			**Exp →Abbr**			**Syn**		
	** RI**	** RP**	**Result**	** RI**	** RP**	**Result**	** RI**	** RP**	**Result**
*RI* ⊂ *RP*_30_	RI_8	RP_8_sw	0.38	RI_8	RP_8	0.30	RI_8	RP_8	0.39
				RI_4	RP_4_sw	0.30	RI_8	RP_8	0.38
*RP* ⊂ *RI*_30_	RI_20	RP_4_sw	0.35	RI_20	RP_4_sw		RI_8	RP_8_sw	
							RI_20	RP_2_sw	
*RI* + *RP*	RI_4	RP_4_sw	**0.42**	RI_4	RP_4_sw	**0.32**	RI_20	RP_4_sw	**0.40**
				RI_8	RP_8_sw				

Results (recall, top ten) of the best configurations for each model and model combination on the three tasks. The configurations are described according to the following pattern: *model_windowSize*. For RP, *sw* means that stop words are retained in the model.

Using the semantic spaces induced from the medical corpus, again, the *RI*+*RP* combination strategy outperforms the *RI*⊂*RP*_30_ and *RP*⊂*RI*_30_ strategies: 0.10 recall on the *abbr* →*exp* task, 0.08 recall on the *exp* →*abbr* task, and 0.30 recall on the *syn* task (Table 5) are obtained. This combination outperforms the other two by a large margin on the *exp* →*abbr* task: 0.08 recall compared to 0.03 recall. The most appropriate window sizes for capturing these phenomena in the medical corpus are fairly similar to those that worked best with the clinical corpus. On the *abbr* →*exp* task, the optimal window sizes are indeed identical across the two corpora: a 2 + 2 context window with an RP space that incorporates stop words yields the highest performance. For the *exp* →*abbr* task, a slightly larger context window of 4 + 4 seems to work well – again, with stop words retained in the RP space. Alternatively, combining a large RI space (10 + 10) with a smaller RP space (2 + 2, with stop words) performs comparably on this task and with this test data. Finally, for synonyms, a large RI space (10 + 10) with a very small RP space (1 + 1) that retains all words best captures this phenomenon with this type of corpus.

**Table 5:** Results on medical development set

**Strategy**	**Abbr →Exp**			**Exp →Abbr**			**Syn**		
	** RI**	** RP**	**Result**	** RI**	** RP**	**Result**	** RI**	** RP**	**Result**
*RI* ⊂ *RP*_30_	RI_4	RP_4_sw	0.08	RI_2	RP_2	0.03	RI_20	RP_4_sw	0.26
	RI_20	RP_2		RI_4	RP_4				
	RI_20	RP_4_sw		RI_4	RP_4_sw				
				RI_8	RP_8				
				RI_20	RP_2				
				RI_20	RP_2_sw				
				RI_20	RP_4				
				RI_20	RP_4_sw				
*RP* ⊂ *RI*_30_	RI_2	RP_2_sw	0.08	RI_2	RP_2	0.03	RI_8	RP_8_sw	0.24
	RI_4	RP_4		RI_2	RP_2_sw				
	RI_4	RP_4_sw		RI_4	RP_4				
	RI_8	RP_8		RI_4	RP_4_sw				
	RI_8	RP_8_sw		RI_8	RP_8				
	RI_20	RP_2_sw		RI_8	RP_8_sw				
	RI_20	RP_4		RI_20	RP_2				
	RI_20	RP_4_sw		RI_20	RP_2_sw				
				RI_20	RP_4				
				RI_20	RP_4_sw				
*RI* + *RP*	RI_4	RP_4_sw	**0.10**	RI_8	RP_8_sw	**0.08**	RI_20	RP_2_sw	**0.30**
				RI_20	RP_4_sw				

Results (recall, top ten) of the best configurations for each model and model combination on the three tasks. The configurations are described according to the following pattern: *model_windowSize*. For RP, *sw* means that stop words are retained in the model.

Using the semantic spaces induced from the conjoint corpus, the *RI*⊂*RP*_30_ combination strategy outperforms the other two strategies on the abbr →exp task: 0.30 recall compared to 0.25 and 0.23 (Table 6). On the exp →abbr task, this and the *RI*+*RP* combination strategy perform equally well, with 0.18 recall. Finally, on the synonym task, the *RI*+*RP* performs best with a recall of 0.46. In general, somewhat larger window sizes seem to work better when combining semantic spaces induced from the conjoint corpus.

**Table 6:** Conjoined corpus space results on clinical + medical development set

**Strategy**	**Abbr →Exp**			**Exp →Abbr**			**Syn**		
	**RI**	** RP**	**Result**	**RI**	**RP**	**Result**	**RI**	**RP**	**Result**
*RI* ⊂ *RP*_30_	RI_4	RP_4_sw	**0.30**	RI_4	RP_4_sw	**0.18**	RI_8	RP_8_sw	0.41
	RI_20	RP_4_sw							
*RP* ⊂ *RI*_30_	RI_4	RP_4	0.23	RI_4	RP_4_sw	0.13	RI_8	RP_8	0.36
	RI_4	RP_4_sw		RI_8	RP_8_sw		RI_8	RP_8_sw	
	RI_8	RP_8		RI_20	RP_2_sw		RI_20	RP_2_sw	
	RI_20	RP_2		RI_20	RP_4_sw		RI_20	RP_4_sw	
	RI_20	RP_4							
*RI* + *RP*	RI_2	RP_2_sw	0.25	RI_4	RP_4_sw	**0.18**	RI_8	RP_8_sw	**0.46**
				RI_8	RP_8_sw				
				RI_20	RP_4_sw				

Results (recall, top ten) of the best configurations for each model and model combination on the three tasks. The configurations are described according to the following pattern: *model_windowSize*. For RP, *sw* means that stop words are retained in the model.

The best-performing combinations from each corpus and for each task were then treated as (ensemble) baselines in the final evaluation, where combinations of semantic spaces from multiple corpora are evaluated.

### Combination strategies: multiple corpora

The pair of semantic spaces from each corpus that performed best on the three tasks were subsequently employed in combinations that involved four semantic spaces – two from each corpus: one RI space and one RP space. The single-step approaches generally performed better than the two-step approaches, with some exceptions (Table 7). The most successful ensemble was a simple single-step approach, where the cosine similarity scores produced by each semantic space were simply summed (*SUM*), yielding 0.32 recall for *abbr* →*exp*, 0.17 recall for *exp* →*abbr*, and 0.52 recall for *syn*. The *AVG* option, although the second-highest performer on the abbreviation-expansion tasks, yielded significantly poorer results. Normalization, whereby ranking was used instead of cosine similarity, invariably affected performance negatively, especially when employed in conjunction with *SUM*. The two-step approaches performed significantly worse than all non-normalized single-step approaches, with the sole exception taking place on the synonym extraction task. It should be noted that normalization was always performed in the two-step approaches – this was done after each pair of semantic spaces from a single corpus had been combined. Of the four two-step combination strategies, *AVG* →*AVG* and *AVG* →*SUM* performed best, with identical recall scores on the three tasks.

**Table 7:** Disjoint corpus ensemble results on clinical + medical development set

** Strategy**	**Normalize**	**Abbr →Exp**		**Exp →Abbr**		**Syn**	
		** Clinical**	** Medical**	** Clinical**	** Medical**	** Clinical**	** Medical**
		***RI_4***	***RI_4***	***RI_4***	***RI_8***	***RI_20***	***RI_20***
		***RP_4_sw***	***RP_4_sw***	***RP_4_sw***	***RP_8_sw***	***RP_4_sw***	***RP_2_sw***
AVG	*True*	0.13		0.09		0.39	
AVG	*False*	0.24		0.11		0.39	
SUM	*True*	0.13		0.09		0.34	
SUM	*False*	**0.32**		**0.17**		**0.52**	
AVG →AVG		0.15		0.09		0.41	
SUM →SUM		0.13		0.07		0.40	
AVG →SUM		0.15		0.09		0.41	
SUM →AVG		0.13		0.07		0.40	

Results (P = weighted precision, R = recall, top ten) of the best models with and without post-processing on the three tasks. Dynamic # of suggestions allows the model to suggest less than ten terms in order to improve precision. The results are based on the application of the model combinations to the development data.

### Final evaluations

The combination strategies that performed best on the development sets were finally evaluated on completely unseen data in order to assess their generalizability to new data and to assess their expected performance in a deployment setting. Each evaluation phase involves comparing the results to one or more baselines: in the case of single-corpus combinations, the comparisons are made to RI and RP in isolation; in the case of multiple-corpora combinations, the comparisons are made to semantic spaces induced from a single corpus (as well as the conjoint corpus) and ensembles of semantic spaces induced from a single corpus (and, again, the conjoint corpus).

When applying the single-corpus combinations from the clinical corpus, the following results were obtained: 0.31 recall on *abbr* →*exp*, 0.20 recall on *exp* →*abbr*, and 0.44 recall on *syn* (Table 8). Compared to the results on the development sets, the results on the two abbreviation-expansion tasks decreased by approximately ten percentage points; on the synonym extraction task, the performance increased by a couple of percentage points. The RI baseline was outperformed on all three tasks; the RP baseline was outperformed on two out of three tasks, with the exception of the *exp* →*abbr* task. Finally, it might be interesting to point out that the RP baseline performed better than the RI baseline on the two abbreviation-expansion tasks, but that the RI baseline did somewhat better on the synonym extraction task.

**Table 8:** Results on clinical evaluation set

**Evaluation configuration**	**Abbr →Exp**		**Exp →Abbr**		**Syn**	
	***RI_4+RP_4_sw***		***RI_4+RP_4_sw***		***RI_20+RP_4_sw***	
	**P**	**R**	**P**	**R**	**P**	**R**
RI Baseline	0.04	0.22	0.03	0.19	0.07	0.39
RP Baseline	0.04	0.23	0.04	0.24	0.06	0.36
Clinical Ensemble	0.05	0.31	0.03	0.20	0.07	**0.44**
+Post-Processing (Top 10)	0.08	**0.42**	0.05	**0.33**	**0.08**	0.43
+Dynamic Cut-Off (Top ≤ 10)	**0.11**	0.41	**0.12**	0.33	0.08	0.42

Results (P = weighted precision, R = recall, top ten) of the best models with and without post-processing on the three tasks. Dynamic # of suggestions allows the model to suggest less than ten terms in order to improve precision. The results are based on the application of the model combinations to the evaluation data. The improvements in recall between the best baseline and the ensemble method for the synonym task and for the abbr →exp task are both statistically significant for a p-value < 0.05. (abbr →exp task: p-value = 0.022 and synonym task: p-value = 0.002.) The improvement in recall that was achieved by post-processing is statistically significant for both abbreviation tasks (p-value = 0.001 for abbr →exp and p-value = 0.000 for exp →abbr).

With the medical corpus, the following results were obtained: 0.17 recall on *abbr* →*exp*, 0.11 recall on *exp* →*abbr*, and 0.34 recall on *syn* (Table 9). Compared to the results on the development sets, the results were higher for all three tasks. Both the RI and RP baselines were outperformed, with a considerable margin, by their combination. However, the improvement in recall for the combination method compared to the best baseline was only statistically significant for the synonym task. In complete contrast to the clinical corpus, the RI baseline here outperformed the RP baseline on the two abbreviation-expansion tasks, but was outperformed by the RP baseline on the synonym extraction task.

**Table 9:** Results on medical evaluation set

**Evaluation configuration**	**Abbr →Exp**		**Exp →Abbr**		**Syn**	
	***RI_4+RP_4_sw***		***RI_8+RP_8_sw***		***RI_20+RP_2_sw***	
	**P**	**R**	**P**	**R**	**P**	**R**
RI baseline	0.02	0.09	0.01	0.08	0.03	0.18
RP baseline	0.01	0.06	0.01	0.05	0.05	0.26
Medical ensemble	0.03	**0.17**	0.01	**0.11**	**0.06**	**0.34**
+Post-processing (top 10)	0.03	0.17	0.02	0.11	0.06	0.34
+Dynamic cut-off (top ≤ 10)	**0.17**	0.17	**0.10**	0.11	0.06	0.34

Results (P = weighted precision, R = recall, top ten) of the best semantic spaces with and without post-processing on the three tasks. Dynamic # of suggestions allows the model to suggest less than ten terms in order to improve precision. The results are based on the application of the model combinations to the evaluation data. The difference in recall when using the ensemble method compared to the best baseline is only statistically significant (p-value < 0.05) for the synonym task (p-value = 0.000).

When applying the disjoint corpora ensembles, the following results were obtained on the evaluation sets: 0.30 recall on *abbr* →*exp*, 0.19 recall on *exp* →*abbr*, and 0.47 recall on *syn* (Table 10). Compared to the results on the development sets, the results decreased somewhat on two of the tasks, with *exp* →*abbr* the exception. The p-values for the significance tests of the recall differences in Table 10 are shown in Table 11. The two ensemble baselines were clearly outperformed by the larger ensemble of semantic spaces from two types of corpora on two of the tasks; the clinical ensemble baseline performed equally well on the *exp* →*abbr* task.

**Table 10:** Results on clinical + medical evaluation set

**Evaluation configuration**	**Abbr →Exp**		**Exp →Abbr**		**Syn**	
	** Clinical**	** Medical**	** Clinical**	** Medical**	** Clinical**	** Medical**
	***RI_4***	***RI_4***	***RI_4***	***RI_8***	***RI_20***	***RI_20***
	***RP_4_sw***	***RP_4_sw***	***RP_4_sw***	***RP_8_sw***	***RP_4_sw***	***RP_2_sw***
	**SUM, *False***		**SUM, *False***		**SUM, *False***	
	**P**	**R**	**P**	**R**	**P**	**R**
Clinical space	0.03	0.17	0.03	0.19	0.05	0.29
Medical space	0.01	0.06	0.01	0.08	0.03	0.18
Conjoint corpus space	0.03	0.19	0.01	0.08	0.05	0.30
Clinical ensemble	0.04	0.24	0.03	0.19	0.06	0.34
Medical ensemble	0.02	0.11	0.01	0.11	0.05	0.33
Conjoint corpus ensemble	0.03	0.19	0.02	0.14	0.07	0.40
Disjoint corpora ensemble	0.05	0.30	0.03	0.19	**0.08**	**0.47**
+Post-processing (top 10)	0.07	**0.39**	0.06	**0.33**	0.08	0.47
+Dynamic cut-off (top ≤ 10)	**0.28**	0.39	**0.31**	0.33	0.08	0.45

Results (P = weighted precision, R = recall, top ten) of the best semantic spaces and ensembles on the three tasks. The results are based on the clinical + medical evaluation set and are grouped according to the number of semantic spaces employed: one, two or four. The disjoint corpus ensemble is performed with and without post-processing. A dynamic cut-off allows less than ten terms to be suggested in an attempt to improve precision. Results for tests of statistical significance are shown in Table 11.

**Table 11:** **P-values for recall results presented in Table **10

**P-values, recall**	**Medical**	**Conjoint**	**Clinical**	**Medical**	**Conjoint**	**Disjoint**
**(synonym task)**	**space**	**corpus**	**ensemble**	**ensemble**	**corp. ens.**	**corp. ens.**
Clinical space	**0.011**	1.000	0.057	0.885	**0.003**	**0.000**
Medical space	-	**0.004**	**0.000**	**0.000**	**0.000**	**0.000**
Conjoint corpus	-	-	0.210	1.000	**0.001**	**0.000**
Clinical ensemble	-	-	-	0.480	0.189	**0.001**
Medical ensemble	-	-	-	-	**0.047**	**0.000**
Conjoint corp. ens.	-	-	-	-	-	**0.041**

P-values for the differences between the recall results on the synonym task for the semantic spaces/ensembles presented in Table 10. P-values showing a statistically significant difference (p-value < 0.05) are presented in bold-face.

P-values for the post-processing and for the abbr →exp and exp →abbr are not shown in the table. However, for the significance level p-value < 0.05, there were no statistically significant recall difference between the standard Disjoint Corpora Ensemble and the post-processing version for any of the three tasks (p-value = 0.25 for abbr →exp and p-value = 0.062 for exp →abbr). When testing the recall difference between the pairs of semantic spaces/ensembles shown in Table 10 for the abbr →exp task, there was only a significant difference for the pairs Medical Space vs. Clinical Ensemble (p-value = 0.039), Medical Space vs. Disjoint Corpora Ensemble (p-value = 0.004) and Medical Ensemble vs. Disjoint Corpora Ensemble (p-value = 0.039). For the exp →abbr task, there were no statistically significant differences.

### Post-processing

In an attempt to further improve results, simple post-processing of the candidate terms was performed. In one setting, the system was forced to suggest ten candidate terms regardless of their cosine similarity score or other properties of the terms, such as their length. In another setting, the system had the option of suggesting a dynamic number – ten or less – of candidate terms.

This was unsurprisingly more effective on the two abbreviation-expansion tasks. With the clinical corpus, recall improved substantially with the post-processing filtering: from 0.31 to 0.42 on *abbr* →*exp* and from 0.20 to 0.33 on *exp* →*abbr* (Table 8). With the medical corpus, however, almost no improvements were observed for these tasks (Table 9). For the combination of semantic spaces from the two corpora, the improvements in recall after applying post-processing on the two abbreviation tasks are not statistically significant (Table 10).

With a dynamic cut-off, only precision could be improved, although at the risk of negatively affecting recall. With the clinical corpus, recall was largely unaffected for the two abbreviation-expansion task, while precision improved by 3–7 percentage points (Table 8). With the medical corpus, the gains were even more substantial: from 0.03 to 0.17 precision on *abbr* →*exp* and from 0.02 to 0.10 precision on *exp* →*abbr* – without having any impact on recall (Table 9). The greatest improvements on these tasks were, however, observed with the combination of semantic spaces from multiple corpora: precision increased from 0.07 to 0.28 on *abbr* →*exp* and from 0.06 to 0.31 on *exp* →*abbr* – again, without affecting recall (Table 10).

In the case of synonyms, this form of post-processing is more challenging, as there are no simple properties of the terms, such as their length, that can serve as indications of their quality as candidate synonyms. Instead, one has to rely on their use in different contexts and grammatical properties; as a result, cosine similarity and ranking of the candidate terms were exploited in an attempt to improve the candidate synonyms. This approach was, however, clearly unsuccessful for both corpora and their combination, with almost no impact on either precision or recall. In a single instance – with the clinical corpus – precision increased by one percentage point, albeit at the expense of recall, which suffered a comparable decrease (Table 8). With the combination of semantic spaces from two corpora, the dynamic cut-off option resulted in a lower recall score, without improving precision (Table 10).

### Frequency thresholds

**Figure 5:**
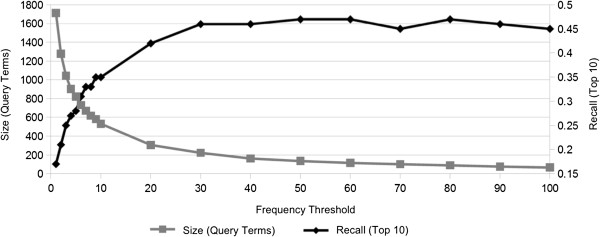
**Frequency thresholds.** The relation between recall and the required minimum frequency of occurrence for the reference standard terms in both corpora. The number of query terms for each threshold value is also shown.

In order to study the impact of different frequency thresholds – i.e., how often each pair of terms had to occur in the corpora to be included in the reference standard – on the task of extracting synonyms, the best ensemble system was applied to a range of evaluation sets with different thresholds from 1 to 100 (Figure 5). With a low frequency threshold, it is clear that a lower performance is obtained. For instance, if each synonym pair only needs to occur at least once in both corpora, a recall of 0.17 is obtained. As the threshold is increased, recall increases too - up to a frequency threshold of around 50, after which no performance boosts are observed. Already with a frequency threshold of around 30, the results seem to level off. With frequency thresholds over 100, there is not enough data in this case to produce any reliable results.

## Discussion

The results clearly demonstrate that combinations of semantic spaces lead to improved results on the synonym extraction task. For the two abbreviation tasks, most of the observed performance gains were not statistically significant. Combining random indexing and random permutation allows slightly different aspects of lexical semantics to be captured; by combining them, stronger semantic relations between terms are extracted, thereby increasing the performance on these tasks. Combining semantic spaces induced from different corpora further improves performance. This demonstrates the potential of distributional ensemble methods, of which this – to the extent of our knowledge – is the primary implementation of its kind, and it only scratches the surface. In this initial study, only four semantic spaces were used; however, with increasing computational capabilities, there is nothing stopping a much larger number of semantic spaces from being combined. These can capture various aspects of semantics – aspects which may be difficult, if not impossible, to incorporate into a single model – from a large variety of observational data on language use, where the contexts may be very different.

### Clinical vs. medical corpora

When employing corpus-driven methods to support lexical resource development, one naturally needs to have access to a corpus in the target domain that reflects the language use one wishes to model. Hence, one cannot, without due qualification, state that one corpus type is better than another for the extraction of synonyms or abbreviation-expansion pairs. This is something that needs to be duly considered when comparing the results for the semantic spaces on the clinical and medical corpora, respectively. Another issue concerns the size of each corpus: in fact, the size of the medical corpus is only half as large as the clinical corpus (Table 1). The reference standards used in the respective experiments are, however, not identical: each term pair had to occur at least fifty times to be included – this will differ across corpora. To some extent this mitigates the effect of the total corpus size and makes the comparison between the two corpora fairer; however, differences in reference standards also entail that the results presented in Tables 8 and 9 are not directly comparable. Another difference between the two corpora is that the clinical corpus contains more unique terms (word types) than the medical corpus, which might indicate that it consists of a larger number of concepts. It has previously been shown that it can be beneficial, indeed important, to employ a larger dimensionality when using corpora with a large vocabulary, as is typically the case in the clinical domain [57]; in this study a dimensionality of 1,000 was used to induce all semantic spaces. The results, on the contrary, seem to indicate that better performance is generally obtained with the semantic spaces induced from the clinical corpus.

An advantage of using non-sensitive corpora like the medical corpus employed in this study is that they are generally more readily obtainable than sensitive clinical data. Perhaps such and similar sources can complement smaller clinical corpora and yet obtain similar or potentially even better results.

### Combining semantic spaces

Creating ensembles of semantic spaces has been shown to be profitable, at least on the task of extracting synonyms and abbreviation-expansion pairs. In this study, the focus has been on combining the *output* of the semantic spaces. This is probably the most straightforward approach and it has several advantages. For one, the manner in which the semantic representations are created can largely be ignored, which would potentially allow one to combine models that are very different in nature, as long as one can retrieve a ranked list of semantically related terms with a measure of the strength of the relation. It also means that one can readily combine semantic spaces that have been induced with different parameter settings, for instance with different context definitions and of different dimensionality. An alternative approach would perhaps be to combine semantic spaces on a *vector level*. Such an approach would be interesting to explore; however, it would pose numerous challenges, not least in combining context vectors that have been constructed differently and potentially represent meaning in disparate ways.

Several combination strategies were designed and evaluated. In both the single-corpus and multiple-corpora ensembles, the most simple strategy performed best: the one whereby the cosine similarity scores are summed. There are potential problems with such a strategy, since the similarity scores are not absolute measures of semantic relatedness, but merely relative and only valid within a single semantic space. The cosine similarity scores will, for instance, differ depending on the distributional model used and the size of the context window. An attempt was made to deal with this by replacing the cosine similarity scores with ranking information, as a means to *normalize* the output of each semantic space before combing them. This approach, however, yielded much poorer results. A possible explanation for this is that a measure of the semantic relatedness between terms is of much more importance than their ranking. After all, a list of the highest ranked terms does not necessarily imply that they are semantically similar to the query term; only that they are the most semantically similar in this space. For the multiple-corpora ensembles, the AVG strategy was applied with the aim of not penalizing candidate synonyms that only appear in one of the two corpora. It is not surprising that this strategy was not successful given the form of the evaluation, which consisted of suggesting candidate synonyms that were known to occur at least 50 times in both corpora. The two-step approaches for the multiple-corpora ensembles all included a normalizing and/or averaging component, resulting in a lower recall compared to the SUM strategy, probably for the same reasons as when these strategies were applied in the one-step approach.

To gain deeper insights into the process of combining the output of multiple semantic spaces, an error analysis was conducted on the synonym extraction task. This was achieved by comparing the outputs of the most profitable combination of semantic spaces from each corpus, as well as with the combination of semantic spaces from the two corpora. The error analysis was conducted on the development sets. Of the 68 synonyms that were correctly identified as such by the corpora combination, five were not extracted by either of the single-corpus combinations; nine were extracted by the medical ensemble but not by the clinical ensemble; as many as 51 were extracted by the clinical ensemble but not by its medical counterpart; in the end, this means that only three terms were extracted by both the clinical and medical ensembles. These results augment the case for multiple-corpora ensembles. There appears to be little overlap in the top-10 outputs of the corpora-specific ensembles; by combining them, 17 additional true synonyms are extracted compared to the clinical ensemble alone. Moreover, the fact that so many synonyms are extracted by the clinical ensemble demonstrates the importance of exploiting clinical corpora and the applicability of distributional semantics to this genre of text. In Table 12, the first two examples, *sjukhem (nursing-home)* and *depression* show cases for which the multiple-corpora ensemble was successful but the single-corpus ensembles were not. In the third example, both the multiple-corpora ensemble and the clinical ensemble extract the expected synonym candidate.

**Table 12:** Examples of extracted candidate synonyms

**Query term: sjukhem *(nursing-home)***		
**Clinical**	**Medical**	**Clinical + Medical**
Heartcenter (*heart-center*)	Vårdcentral (*health-center*)	Vårdcentral (*health-center*)
Bröstklinik (*breast-clinic*)	Akutmottagning (*emergency room*)	Mottagning (*reception*)
Hälsomottagningen (*health-clinic*)	Akuten (*ER*)	**Vårdhem** (*nursing-home*)
Hjärtcenter (*heart-center*)	Mottagning (*reception*)	Gotland (*a Swedish county*)
Län (*county*)	Intensivvårdsavdelning (*ICU*)	Sjukhus (*hospital*)
Eyecenter (*eye-center*)	Arbetsplats (*work-place*)	Gård (*yard*)
Bröstklin (*breast-clin.*)	Vårdavdelning (*ward*)	Vårdavdelning (*ward*)
Sjukhems (*nursing-home’s*)	Gotland (*a Swedish county*)	Arbetsplats (*work-place*)
Hartcenter (*“hart-center”*)	Kväll (*evening*)	Akutmottagning (*emergency room*)
Biobankscentrum (*biobank-center*)	Ks (*Karolinska hospital*)	Akuten (*ER*)
**Query term: depression *(depression)***		
**Clinical**	**Medical**	**Clinical + Medical**
Sömnstörning (*insomnia*)	Depressioner (*depressions*)	Sömnstörning (*insomnia*)
Sömnsvårigheter (*insomnia*)	Osteoporos (*osteoporosis*)	Osteoporos (*osteoporosis*)
Panikångest (*panic disorder*)	Astma (*asthma*)	Tvångssyndrom (*OCD*)
Tvångssyndrom (*OCD*)	Fetma (*obesity*)	Epilepsi (*epilepsy*)
Fibromyalgi (*fibromyalgia*)	Smärta (*pain*)	Hjärtsvikt (*heart failure*)
Ryggvärk (*back-pain*)	Depressionssjukdom (*depressive-illness*)	**Nedstämdhet** (*sadness*)
Självskadebeteende (*self-harm*)	Bensodiazepiner (*benzodiazepines*)	Fibromyalgi (*fibromyalgia*)
Osteoporos (*osteoporosis*)	Hjärtsvikt (*heart-failure*)	Astma (*asthma*)
Depressivitet (*“depressitivity”*)	Hypertoni (*hypertension*)	Alkoholberoende (*alcoholism*)
Pneumoni (*pneumonia*)	Utbrändhet (*burnout*)	Migrän (*migraine*)
**Query term: allergi *(allergy)***		
**Clinical**	**Medical**	**Clinical + Medical**
Pollenallergi (*pollen-allergy*)	Allergier (*allergies*)	Allergier (*allergies*)
Födoämnesallergi (*food-allergy*)	Sensibilisering (*sensitization*)	Hösnuva (*hay-fever*)
Hösnuva (*hay-fever*)	Hösnuva (*hay-fever*)	Födoämnesallergi (*food-allergy*)
**Överkänslighet** (*hypersensitivity*)	Rehabilitering (*rehabilitation*)	Pollenallergi (*pollen-allergy*)
Kattallergi (*cat-allergy*)	Fetma (*obesity*)	**Överkänslighet** (*hypersensitivity*)
Jordnötsallergi (*peanut-allergy*)	Kol (*COPD*)	Astma (*asthma*)
Pälsdjursallergi (*animal-allergy*)	Osteoporos (*osteoporosis*)	Kol (*COPD*)
Negeras (*negated*)	Födoämnesallergi (*food-allergy*)	Osteoporos (*osteoporosis*)
Pollen (*pollen*)	Astma (*asthma*)	Jordnötsallergi (*peanut-allergy*)
Pollenallergiker (*“pollen-allergic”*)	Utbrändhet (*burnout*)	Pälsdjursallergi (*animal-allergy*)

The top ten candidate synonyms for three different query terms with the clinical ensemble, the medical ensemble and the disjoint corpus ensemble. The synonym in the reference standard is in boldface.

There was one query term – the drug name *omeprazol* – for which both single-corpus ensembles were able to identify the synonym, but where the multiple-corpora ensemble failed. There were also three query terms for which synonyms were identified by the clinical ensemble, but not by the multiple-corpora ensemble; there were five query terms that were identified by the medical ensemble, but not by the multiple-corpora ensemble. This shows that combining semantic spaces can also, in some cases, introduce noise.

Since synonym pairs were queried both ways, i.e. each term in the pair would be queried to see if the other could be identified, we wanted to see if there were cases where the choice of query term would be important. Indeed, among the sixty query terms for which the expected synonym was not extracted, this was the case in fourteen instances. For example, given the query term *blindtarmsinflammation (“appendix-inflammation”)*, the expected synonym *appendicit (appendicitis)* was given as a candidate, whereas with the query term *appendicit*, the expected synonym was not successfully identified.

Models of distributional semantics face the problem of modeling terms with several ambiguous meanings. This is, for instance, the case with the polysemous term *arv* (referring to *inheritance* as well as to *heredity*). Distant synonyms also seem to be problematic, e.g. the pair *rehabilitation*/*habilitation*. For approximately a third of the synonym pairs that are not correctly identified, however, it is not evident that they belong to either of these two categories.

### Post-processing

In an attempt to improve results further, an additional step in the proposed method was introduced: filtering of the candidate terms, with the possibility of extracting new, potentially better ones. For the extraction of abbreviation-expansion pairs, this was fairly straightforward, as there are certain patterns that generally apply to this phenomenon, such as the fact that the letters in an abbreviation are contained – in the same order – in its expansion. Moreover, expansions are longer than abbreviations. This allowed us to construct simple yet effective rules for filtering out unlikely candidate terms for these two tasks. As a result, both precision and recall increased; with a dynamic cut-off, precision improved significantly. Although our focus in this study was primarily on maximizing recall, there is a clear incentive to improve precision as well. If this method were to be used for terminological development support, with humans inspecting the candidate terms, minimizing the number of poor candidate terms has a clear value. However, given the seemingly easy task of filter out unlikely candidates, it is perhaps more surprising that the results were not even better. A part of the reason for this may stem from the problem of semantically overloaded word types, which affects abbreviations to a large degree, particularly in the clinical domain with its telegraphic style and where ad-hoc abbreviations abound. This was also reflected in the reference standard, as in some cases the most common expansion of an abbreviation was not included.

The post-processing filtering of synonyms clearly failed. Although ranking information and, especially, cosine similarity provide some indication of the quality of synonym candidates, employing cut-off values with these features can impossibly improve recall: new candidates will always have a lower ranking and a lower cosine similarity score than discarded candidate terms. It can, however – at least in theory – potentially improve precision when using these rules in conjunction with a dynamic cut-off, i.e. allowing less than ten candidates terms to be suggested. In this case, however, the rules did not have this effect.

### Thresholds

Increasing the frequency threshold further did not improve results. In fact, a threshold of 30 occurrences in both corpora seems to be sufficient. A high frequency threshold is a limitation of distributional methods; thus, the ability to use a lower threshold is important, especially in the clinical domain where access to data is difficult to obtain.

The choice of evaluating recall among ten candidates was based on an estimation of the number of candidate terms that would be reasonable to present to a lexicographer for manual inspection. Recall might improve if more candidates were presented, but it would likely come at the expense of decreased usability. It might instead be more relevant to limit further the number of candidates to present. As is shown in Figure 4, there are only a few correct synonyms among the candidates ranked 6–10. By using more advanced post-processing techniques and/or being prepared to sacrifice recall slightly, it is possible to present fewer candidates for manual inspection, thereby potentially increasing usability. On the other hand, a higher cut-off value could be used for evaluating a system aimed at a user who is willing to review a longer list of suggestions. An option for incorporating this difference in user behavior would be to use an evaluation metrics, such as rank-biased precision [58], that models the persistence of the user in examining additional lower-ranked candidates.

### Reflections on evaluation

To make it feasible to compare a large number of semantic spaces and their various combinations, fixed reference standards derived from terminological resources were used for evaluation, instead of manual classification of candidate terms. One of the motivations for the current study, however, is that terminological resources are seldom complete; they may also reflect a *desired* use of language rather than *actual* use. A manual classification on a sample of one of the reference standards, *Medical + Clinical*, was carried out on the synonym task in order to verify this claim. The results in this study thus mainly reflect to what extent different semantic spaces – and their combinations – are able to extract synonymous relations that have been considered relevant according to specific terminologies, rather than to what extent the semantic spaces – and their combinations – capture the phenomenon of synonymy. This is, for instance, illustrated by the query term *depression* in Table 12, in which one potential synonym is extracted by the clinical ensemble – *depressivitet (“depressitivity”)* – and another potential synonym by the medical ensemble: *depressionsjukdom (depressive illness)*. Although these terms might not be formal or frequent enough to include in all types of terminologies, they are highly relevant candidates for inclusion in terminologies intended for text mining. Neither of these two terms are, however, counted as correct synonyms, and only the multiple-corpora ensemble is able to find the synonym included in the terminology.

Furthermore, a random sample of 30 words (out of 135) was manually classified for the semantic relation between each of the candidate terms in the sample, as suggested by the best combination of semantic spaces (the Disjoint Corpus Ensemble, see Table 10), and the target term. In the reference standard for this sample, 33 synonyms are to be found (only three target words have two synonyms; none have three or more). The best combination finds only 10 of these reference synonyms (exact match), which accounts for the low recall figures in Table 10. However, a manual classification shows that the same combination finds another 29 synonyms that do not occur in the reference standard. Furthermore, the Disjoint Corpus Ensemble also suggests a total of 15 hyponyms, 14 hypernyms and 3 spelling variants as candidate terms, which, depending on the context, can be viewed as synonyms. Among the candidate terms, we also find 3 antonyms, which shows the inability of the models readily to distinguish between different types of semantic relations.

In one instance, we also capture a non-medical sense of a term while completely missing the medical sense. For the target term *sänka* (erythrocyte sedimentation rate), 9 out of 10 candidate terms relate to the more general sense of lowering something (also *sänka* in Swedish), with candidate terms such as *rising, reducing, increasing, halving* and *decreasing*. None of these are included in the reference standard, which for this word only contains the abbreviation *SR* (ESR) as a synonym.

In the case of the target term *variecella*, the reference standard contains only the synonym *vattkoppor* (chickenpox), while the Disjoint Corpus Ensemble correctly suggests the abbreviation *VZV*, as well as *herpes* and the plural form *varicellae* (which is apparently missed by the lemmatizer).

It is important to recognize that this type of manual post-evaluation always bears the risk that you are too generous, believing in your method, and thus (manually) assign too many correct classifications – or, alternatively that you are too strict in your classification in fear of being too generous. Future studies would thus benefit from an extensive manual classification of candidates derived from data generated in clinical practice, beforehand, with the aim of also finding synonyms that are not already included in current terminologies but are in frequent use. These could then be used as reference standards in future evaluations.

The choice of terminological resources to use as reference standards was originally based on their appropriateness for evaluating semantic spaces induced from the clinical corpus. However, for evaluating the extraction of abbreviation-expansion pairs with semantic spaces induced from the medical corpus, the chosen resources – in conjunction with the requirement that terms should occur at least fifty times in the corpus – were less appropriate, as it resulted in a very small reference standard. This, in turn, resulted in no significant differences for either of the two the abbreviation tasks between the best single space and the combination of medical spaces, or between the conjoint corpus ensemble and the disjoint corpus ensemble. When assessing the potential of using semantic spaces for abbreviation-expansion tasks, more focus should therefore be put on the results from the evaluation on the spaces created from the clinical corpus, as the improvement in recall gained by post-processing was statistically significant for both the abbr →exp task and the exp →abbr task, as was also the improvement gained from using an ensemble of spaces compared to a single corpus space for the abbr →exp task.

For synonyms, the number of instances in the reference standard is, of course, smaller for the experiments with multiple-corpora ensembles than for the single-corpus experiments. However, the differences between the single space and the ensemble of spaces are statistically significant. Moreover, when evaluating the final results with different frequency thresholds, similar results are obtained when lowering the threshold and, as a result, including more evaluation instances. With a threshold of twenty occurrences, 306 input terms are evaluated, which results in a recall of 0.42; with a threshold of thirty occurrences and 222 query terms, a recall of 0.46 is obtained.

### Future work

Now that this first step has been taken towards creating ensembles of semantic spaces, this notion should be explored in greater depth and taken further. It would, for instance, be interesting to combine a larger number of semantic spaces, possibly including those that have been more explicitly modeled with syntactic information. To verify the superiority of this approach, it should be compared to the performance of a single semantic space that has been induced from multiple corpora.

Further experiments should likewise be conducted with combinations involving a larger number of corpora (types). One could, for instance, combine a professional corpus with a layman corpus – e.g. a corpus of extracts from health-related fora – in order to identify layman expressions for medical terms. This could provide a useful resource for automatic text simplification.

Another technique that could potentially be used to identify term pairs with a higher degree of semantic similarity is to ensure that both terms have each other as their closest neighbors in the semantic subspace. This is not always the case, as we pointed out in our error analysis. This could perhaps improve performance on the task of extracting synonyms and abbreviation-expansion pairs.

A limitation of the current study – in the endeavor to create a method that accounts for the problem of language use variability – is that the semantic spaces were constructed to model only unigrams. Textual instantiations of the same concept can, however, vary in term length. This needs to be accounted for in a distributional framework and concerns paraphrasing more generally than synonymy in particular. Combining unigram spaces with multiword spaces is a possibility that could be explored. This would also make the method applicable for acronym expansion.

## Conclusions

This study demonstrates that combinations of semantic spaces can yield improved performance on the task of automatically extracting synonyms. First, combining two distributional models – random indexing and random permutation – on a single corpus enables the capturing of different aspects of lexical semantics and effectively increases the quality of the extracted candidate terms, outperforming the use of one model in isolation. Second, combining distributional models and types of corpora – a clinical corpus, comprising health record narratives, and a medical corpus, comprising medical journal articles – improves results further, outperforming ensembles of semantic spaces induced from a single source, as well as single semantic space induced from the conjoint corpus. We hope that this study opens up avenues of exploration for applying the ensemble methodology to distributional semantics.

Semantic spaces can be combined in numerous ways. In this study, the approach was to combine the outputs, i.e. ranked lists of semantically related terms to a given query term, of the semantic spaces. How this should be done is not wholly intuitive. By exploring a variety of combination strategies, we found that the best results were achieved by simply summing the cosine similarity scores provided by the distributional models.

On the task of extracting abbreviation-expansion pairs, substantial performance gains were obtained by applying a number of simple post-processing rules to the list of candidate terms. By filtering out unlikely candidates based on simple patterns and retrieving new ones, both recall and precision were improved by a large margin.

Lastly, analysis of a manually classified sample from the synonym task shows that the semantic spaces not only extract synonyms that are present in the reference standard. Equally valid synonyms not present in the reference standard are also found. This serves to show that the reference standards, as most often is the case, lack in coverage, as well as supports the fact that the semantic spaces can be used to enrich and expand such resources.

## Endnotes

^a^ Signifiers are here simply different linguistic items referring to the same concept.

^b^ Ontologies are formal descriptions of concepts and their relationships.

^c^ The words *big* and *large* are, for instance, synonymous when describing a house, but certainly not when describing a sibling.

^d^ Unified Medical Language System: http://www.nlm.nih.gov/research/umls/

^e^ Hyponyms are words that are subordinate to another word, its hypernym. For instance, *dog* is a hyponym of *mammal*, which in turn is a hyponym of *animal*.

^f^ There are also probabilistic models, which view documents as a mixture of topics and represent terms according to the probability of their occurrence during the discussion of each topic: two terms that share similar topic distributions are assumed to be semantically related.

^g^ Explicit dimensionality reduction is avoided in the sense that an initial term-context matrix is not constructed, the dimensionality of which is then reduced. The high-dimensional data is *prereduced*, if you will, by selecting a much lower dimensionality from the outset (effectively making this a parameter of the model).

^h^ Ternary vectors allow three possible values: +1’s, 0’s and -1’s. Allowing negative vector elements ensures that the entire vector space is utilized.

^i^ Orthogonal index vectors would yield completely uncorrelated context representations; in the RI approximation, *near*-orthogonal index vectors result in almost uncorrelated context representations.

^j^ The bag-of-words model is a simplified representation of a text as an unordered collection of words, where grammar and word order are ignored.

^k^ An alternative is to shift the index vectors according to direction only, effectively producing *direction vectors*[46].

^l^ This research has been approved by the Regional Ethical Review Board in Stockholm (Etikprövningsnämnden i Stockholm), permission number 2012/834-31/5.

^m^ The used stop word lists are available at http://people.dsv.su.se/~mariask/resources/stoppord.txt (clinical corpus) and http://people.dsv.su.se/~mariask/resources/lt_stoppord.txt. (medical corpus)

^n^ Antonyms are words that differ in one dimension of meaning, and thus are mutually exclusive in this sense. For instance, something cannot be both *large* and *small* in size at the same time.

^o^ Hypernyms are words that are superordinate to another word, its hyponym. For instance, *animal* is a hypernym of *mammal*, which in turn is a hypernym of *dog*.

## Competing interests

The authors declare that they have no competing interests.

## Authors’ contributions

AH was responsible for coordinating the study and was thus involved in all parts of it. AH was responsible for the overall design of the study and for carrying out the experiments. AH initiated the idea of combining semantic spaces induced from different corpora and implemented the evaluation and post-processing modules. AH also had the main responsibility for the manuscript and drafted parts of the background and results description. HM and MS contributed equally to the study. HM initiated the idea of combining semantic models trained differently (Random Indexing and Random Permutation) and was responsible for designing and implementing strategies for combining the output of multiple semantic models. HM also drafted parts of the method description in the manuscript and surveyed relevant literature. MS initiated the idea of applying the method to abbreviation-expansion extraction and to different types of corpora. MS was responsible for designing the evaluation part of the study, as well as for preparing the reference standards. MS also drafted parts of the background and method description in the manuscript. VD, together with MS, was responsible for designing the post-processing filtering of candidate terms. MD provided feedback on the design of the study and drafted parts of the background and method description in the manuscript. MD also carried out the manual evaluation, and the analysis thereof. AH, HM, MS and MD analyzed the results and drafted the discussion and conclusions in the manuscript. All authors read and approved the final manuscript.
